# Bringing together the pieces: the need for holistic care for women with HIV

**DOI:** 10.1002/jia2.26228

**Published:** 2024-03-07

**Authors:** Mardge Hillary Cohen, Chantal Benekigeri, Kathryn Anastos

**Affiliations:** ^1^ Department of Medicine Stroger Hospital of Cook County Chicago Illinois USA; ^2^ WE‐ACTx for Hope Kigali Rwanda; ^3^ Departments of Medicine and Epidemiology & Population Health Albert Einstein College of Medicine Bronx New York USA

1

Widespread use of antiretroviral therapy (ART) globally has resulted in dramatic decreases in new HIV acquisitions and markedly lower mortality for people living with HIV (PWH) [1]. Still, in 2022, 630,000 people globally, nearly half of them women, died from AIDS‐related illness, and each week 4000 girls and young women aged 15–24 years acquired HIV. Sub‐Saharan Africa carries the greatest burden of HIV, with 66% of global infections overall, and ∼80% of infections in women [1]. Even those with well‐managed HIV are at continued risk of gender‐based violence (GBV) and increased risk of other preventable and/or treatable medical conditions, including cardiovascular disease (CVD) [2] and cervical cancer [3], both of which disproportionately affect the disadvantaged communities at highest risk for HIV. Overcoming these continuing challenges to ensure optimal clinical outcomes for women with HIV (WWH) requires equitable and enhanced access and resources, with a woman‐determined focus and a human rights perspective.

The overlap between the twin public health epidemics of GBV (30% of women globally experience GBV) and HIV has been well documented for three decades [4, 5, 6]. Recent pooled data from several sub‐Saharan African national surveys affirm the adverse effects of intimate partner violence (IPV) on HIV acquisition and treatment outcomes [7]. Women experiencing physical or sexual violence in the past year were three times more likely to acquire HIV than those who did not report GBV, and WWH who experienced IPV were 9% less likely to be virally suppressed. The authors suggest the impact might be even higher if high‐risk special populations (sex workers, transgender women, younger women) were more fully represented in these studies.

Ending the HIV epidemic, a global public health goal, cannot be achieved without preventing GBV. Integrating HIV care and IPV programmes is critical to addressing the increased transmission and treatment issues associated with IPV [8]. While HIV acquisitions often result from sexual abuse for the individual, other structural factors like gender power inequity and policies also influence both transmission and poor treatment outcomes. Men who abuse women are more likely to have HIV due to disproportionately higher rates of condomless sex and substance use [5]. Integrated HIV/IPV programmes could thus uniquely and powerfully provide safer settings for women to disclose IPV and then engage and support them to continue ART, improving their health outcomes.

Studies on the syndemic of substance use, violence and HIV and other syndemic conditions like food insecurity, poverty, housing instability, stress and mental illness, demonstrate how these co‐occurring conditions reinforce each other, leading to worse health outcomes for WWH [9]. Acknowledging these intersecting issues and implementing effective individual, community and structural interventions has important clinical implications. All healthcare staff should be explicitly and systematically trained to provide culturally adapted trauma‐informed care that is welcoming, safe, non‐stigmatizing and non‐punitive, and addresses the challenging coping strategies of substance use and healthcare avoidance. The specific features of such care should be standardized, measurable and guaranteed. Providing women‐specific economic transfers for food and housing, empowerment programmes, school‐based sexual violence prevention programmes, and evidence‐based medications for alcohol and drug use along with harm reduction and mental health treatment are needed to effectively address these syndemic conditions [10].

WWH are also at higher risk of medical conditions that impact both individual and public health. They have a six‐fold higher risk of cervical cancer than women not living with HIV [3]. The World Health Organization has set a goal of eliminating cervical cancer globally by 2030 [11] through Human Papilloma Virus vaccination for girls and young women, and screening of [unvaccinated] older women (>30 years) with treatment of precancerous lesions. In response, screening programmes are developing worldwide, especially in areas with a high burden of both HIV and cervical cancer (e.g. Sub‐Saharan Africa) and are increasingly integrated into HIV care. These are hopeful developments in our quest to improve the lives of WWH. Yet, we lack some basic information to prevent cervical cancer in WWH: we need further research to determine the effectiveness of HPV vaccination in WWH, including how many doses confer protection, and to define the optimal screening strategies for cervical cancer prevention.

WWH also carry the excess risk of CVD [2], the most common cause of death globally. The adoption of “Western” lifestyles (i.e. higher fat diets, less physical activity) in low‐ and middle‐income countries has led to increasing rates of diabetes, hypertension and CVD [12]. Even well‐treated young PWH have a higher risk of arterial inflammation [2, 13] driven by HIV‐related residual immune activation and inflammation. Traditional cardiac risk scores underperform in PWH, especially in WWH. The REPRIEVE trial [14] demonstrated that in PWH >40 years at low to moderate calculated CV risk, including WWH, statin use prevents adverse CV outcomes. Thus, CVD prevention in PWH requires paradigms of care that go beyond traditional CVD risk assessment. As guidelines for care incorporate these findings, WWH globally will need access to this inexpensive preventive therapy which should be integrated into HIV care settings.

More broadly, resources are needed for interventions to address the full spectrum of the impact of HIV in women. These should include culturally tailored programmes that focus on affordable and accessible high‐quality medical and mental health services, supporting community activism to change harmful gender attitudes and policies, and ending discrimination/criminalization against people who sell sex, people who use drugs, lesbians, bisexual and transgender people [6, 10]. It is unrealistic to expect women to attend separate IPV, substance use, mental health, HIV, general medical, cervical cancer screening and economic transfer programmes. Yet, it is difficult to provide all these services in one setting without making specialists and specialized services available; restructuring and dedicated funding is required. This demands a commitment to a holistic approach where women's needs and preferences for integrated services are prioritized, based on women's input into resource allocation and design of services.

In summary, to achieve the best outcomes for WWH, healthcare providers must continue to partner with WWH to create programmes globally that integrate the multiple structural, medical and life needs of WWH. This is especially difficult in the current political environment in which consistent funding for global programmes is being threatened by stigmatizing and anti‐reproductive justice agendas [15] and domestic US programmes are increasingly designed around an optimal fiscal bottom line at the expense of favourable clinical outcomes [16].

## COMPETING INTERESTS

MHC, CB and KA have no conflicting financial or non‐financial interests to disclose.

## AUTHORS’ CONTRIBUTIONS

MHC, CB and KA contributed to the analysis, writing and review of the manuscript.

## FUNDING

MHC and KA are Principal Investigators in the MACS/WIHS Combined Cohort Study (MWCCS): Chicago‐Cook County CRS (Mardge Cohen and Audrey French), U01‐HL146245; Bronx CRS (Kathryn Anastos, Anjali Sharma and David Hanna), U01‐HL146204. The MWCCS is funded primarily by the National Heart, Lung, and Blood Institute (NHLBI), with additional co‐funding from the Eunice Kennedy Shriver National Institute of Child Health & Human Development (NICHD), National Institute on Aging (NIA), National Institute of Dental & Craniofacial Research (NIDCR), National Institute of Allergy and Infectious Diseases (NIAID), National Institute of Neurological Disorders and Stroke (NINDS), National Institute of Mental Health (NIMH), National Institute on Drug Abuse (NIDA), National Institute of Nursing Research (NINR), National Cancer Institute (NCI), National Institute on Alcohol Abuse and Alcoholism (NIAAA), National Institute on Deafness and Other Communication Disorders (NIDCD), National Institute of Diabetes and Digestive and Kidney Diseases (NIDDK), National Institute on Minority Health and Health Disparities (NIMHD), and in coordination and alignment with the research priorities of the National Institutes of Health, Office of AIDS Research (OAR). KA is a Principal Investigator of Central Africa Epidemiology Databases to Evaluate AIDS (U01‐AI096299) funded by the National Institute of Allergy and Infectious Diseases, the *Eunice Kennedy Shriver* National Institute of Child Health and Human Development, the National Cancer Institute, the National Institute of Mental Health, the National Institute on Drug Abuse, the National Heart, Lung, and Blood Institute, the National Institute on Alcohol Abuse and Alcoholism, the National Institute of Diabetes and Digestive and Kidney Diseases and the Fogarty International Center; and of the Einstein/Rwanda/DRC Consortium for HIV/HPV Malignancies Research (U54‐CA254568) and the Rwanda CASCADE Clinical Trials Site for cervical cancer prevention (1UG1‐CA284908), both funded by the National Cancer Institute.

## DISCLAIMER

The contents of this publication are solely the responsibility of the authors and do not represent the official views of the National Institutes of Health (NIH).
